# The primeval optical evolving matter by optical binding inside and outside the photon beam

**DOI:** 10.1038/s41467-022-33070-w

**Published:** 2022-09-10

**Authors:** Chih-Hao Huang, Boris Louis, Roger Bresolí-Obach, Tetsuhiro Kudo, Rafael Camacho, Ivan G. Scheblykin, Teruki Sugiyama, Johan Hofkens, Hiroshi Masuhara

**Affiliations:** 1 Department of Applied Chemistry, College of Science, National Yang Ming Chiao Tung University, Hsinchu, 30010 Taiwan; 2 Laboratory for Photochemistry and Spectroscopy, Division for Molecular Imaging and Photonics, Department of Chemistry, Katholieke Universiteit Leuven, Leuven, 3001 Belgium; 3 Division of Chemical Physics and NanoLund, Lund University, Lund, 22100 Sweden; 4 Department of Analytical and Applied Chemistry, Institut Químic de Sarrià, Barcelona, 08017 Spain; 5 Laser Science Laboratory, Toyota Technological Institute, Nagoya, 468-8511 Japan; 6 Center for Cellular Imaging, Core Facilities, the Sahlgrenska Academy, University of Gothenburg, Gothenburg, 405 30 Sweden; 7 Graduate School of Materials Science, Nara Institute of Science and Technology, Nara, 630-0192 Japan; 8 Center for Emergent Functional Matter Science, National Yang Ming Chiao Tung University, Hsinchu, 30010 Taiwan; 9 Max Planck Institute for Polymer Research, Mainz, 55128 Germany

**Keywords:** Nanoparticles, Nanophotonics and plasmonics, Optical manipulation and tweezers, Microscopy

## Abstract

Optical binding has recently gained considerable attention because it enables the light-induced assembly of many-body systems; however, this phenomenon has only been described between directly irradiated particles. Here, we demonstrate that optical binding can occur outside the focal spot of a single tightly focused laser beam. By trapping at an interface, we assemble up to three gold nanoparticles with a linear arrangement which fully-occupies the laser focus. The trapping laser is efficiently scattered by this linear alignment and interacts with particles outside the focus area, generating several discrete arc-shape potential wells with a half-wavelength periodicity. Those external nanoparticles inside the arcs show a correlated motion not only with the linear aligned particles, but also between themselves even both are not directly illuminated. We propose that the particles are optically bound outside the focal spot by the back-scattered light and multi-channel light scattering, forming a dynamic optical binding network.

## Abstract (web summary)

Optical binding enables light-induced assembly of many particles within a focus area. Here, the authors demonstrate that optical binding can occur outside the irradiated area by scattered light interacting with the particles outside the focus, generating arc-shape potential wells for particle trapping.

## Introduction

The many-body problem is a general name for a vast category of physical problems pertaining to the properties of (microscopic) systems made of many interacting particles and as such it is a fundamental physical problem governing nature^1^. From the macroscopic universe of the galaxy to the microscopic world and quantum mechanics, different kinds of matter are complexly bound with each other through different types of interactions. On the one side, we have the planets bound simultaneously to satellite and stars through gravity. On the other side, we have electrons, which can interact with each other through electron exchange while being bound to the nucleus. Between both size limits, there are other systems where the many-body problem applies. Among them, optical binding has attracted the attention of the scientific community because many nano- and micro-scale objects can be bound with each other forming complex self-assembling structures through photon exchange^2^.

Since 1986, optical trapping (optical tweezers) has been used in various research fields (e.g., biology, chemistry, physics, and material sciences) for three-dimensional trapping and manipulation of micro- and nano-scale objects (e.g., nanoparticles (NPs), live cells, proteins, DNA, or small molecules)^3–7^. Upon irradiation with a tightly focused laser beam, these objects are attracted to the focus due to the spatial gradient of the laser field (gradient force). In addition, they are pushed along the propagation direction of light due to photon momentum transfer (scattering and absorption forces). Inside a bulk solution, these objects are only stably trapped at the laser focus when the gradient force is larger than the scattering force^8,9^. At an interface such as a glass/solution interface (as also shown in the present study), all the optical forces contribute to stably trapping and assembling the objects.

In 1989, Burns et al. optically trapped two polystyrene microparticles in a laser beam. They found that the stability of the system raised when the particles were separated by a discrete distance, equal to a multiple of the laser wavelength^2^. Under this optical condition, both particles mutually interacted via scattered light, leading to an interparticle optical force called optical binding force. Like ordinary materials, organized by the electron exchange interaction forming chemical bonds, optically bound particles, optical matter, are organized by the photon exchange interaction^10^. Following the work of Burns et al., several particle configurations have been reported for different optical field geometries^11^. Examples include the formation of a straight alignment (chain-like) of polymer microparticles by counter-propagating laser beams^12,13^; or the formation of different hexagonal structures of polymer NPs by evanescent waves and their polarization control^14–16^. The configurations of these particles depend on the total light field where the incident light field is modified by multiple scatterings from the assembled particles inside the directly irradiated area.

Historically, optical binding has been studied using microparticles, whose volume is large enough to induce strong light scattering, which leads to observable optical binding phenomena. Recently, metallic NPs have been postulated as an alternative to the dielectric microparticles due to their inherent surface plasmon resonance properties, which enhances their light scattering efficiency^17–19^. Concretely, their dynamics has been described and rationalized either experimentally or theoretically inside the irradiated area by changing the intensity, the spatial profile, the phase gradient, and the polarization of the incident laser^20–24^. For example, a collimated circular polarized laser beam (~13 μm in diameter) was used to assemble 101 Au NPs, yielding a hexagonal structure with an interparticle distance equal to the trapping wavelength at the glass/solution interface^25^. These optically-binding induced assemblies, which gather a large number of particles, result from the large irradiated area by the trapping laser. To the best of our knowledge, the optical binding phenomenon so far has only been reported inside the irradiated area.

On the other hand, we had previously reported that a large assembly of 200 nm Au NPs with dynamically fluctuating swarms was formed outside the focus by tightly focusing a laser beam at the glass/solution interface^26–28^. Briefly, during the initial stages, an antenna-like structure with wavelength periodicity was formed inside the focus due to the optical binding. Upon increasing the NP number, this structure was further expanded outside the focus, forming a dumbbell-shaped swarming assembly, which extends up to several tens of micrometers. We proposed that the trapping laser was efficiently scattered by the antenna-like structure toward the outside, consequently trapping more NPs, which self-assembled outside the focal spot.

Here, we present clear experimental evidence that the NPs outside the focus are also optically bound with the NPs inside the focus. The NP dynamics have been fully resolved by single-particle tracking (SPT), revealing different aspects from the well-established optical binding dynamics inside the laser focus. For instance, the external NPs are localized outside the focus in discrete arc-shaped distributions with a half-wavelength periodicity, resembling orbiting electrons populated in quantized energy levels around nuclei. These external and internal NPs interacted with each other even if direct laser irradiation was not applied to external ones. As the electrons bound to the nucleus can exist only in certain quantized orbits where their de Broglie waves are constructively interfering, the present many-body system where each NP is optically bound through light scattering also shows a unique discrete arc-shaped distribution based on the wave properties of photons.

## Results

### Optimization of the optical and material conditions to achieve strong optical binding

**Fig. 1:**
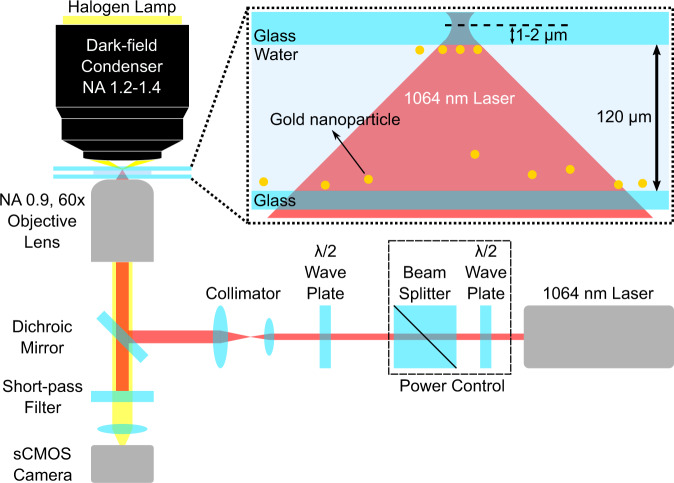
Experimental setup for optical trapping of Au NPs at the upper glass/solution interface. The upper dashed-line inset shows the illustration of the sample conditions at the trapping spot.

A 1064 nm continuous wave laser is tightly focused at approximately 1–2 μm inside the upper glass/solution interface to trap the Au NPs (Fig. 1; further details about the optical setup and sample preparation are described in Methods). The motion of the trapped particles is tracked using a house-written SPT algorithm, which is described in Supplementary Information S1 and available at https://github.com/BorisLouis/goldTracking/^29^.

We selected the following experimental conditions to ensure a sufficient strong optical binding force. First, optical binding strength scales with the NP polarizability, which in its turn scales with the volume of the NP, especially for Rayleigh-sized scatterers^19^. Therefore, we simply use larger Au NPs (400 nm diameter) to maximize the NP’s polarizability. We used a commercial highly monodisperse (<12% variability in size and shape) spherical Au NPs to ensure that all of them will behave similarly. Second, we use linearly polarized light conditions for the trapping laser. The light is scattered to the direction perpendicular to linear polarization, and the external NPs are mainly distributed perpendicular to the direction of linear polarization, which restricts the complex particle motion to a single dimension. Third, we use a highly diluted Au NPs suspension (1.9 × 10^5^ particles/mL) to control the number of trapped particles in the experiment timescale. As a control, we increased 200-fold the Au NPs suspension concentration, and we observed the formation of the dumbbell-shaped swarming assembly (See Supplementary Information S2) as previously reported in our work using smaller Au NPs^26^.

### One to three-nanoparticle systems

**Fig. 2:**
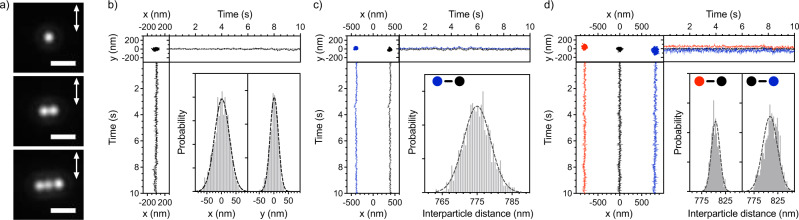
One to three Au NPs systems. **a** Representative images for the three studied systems. The white scale bar is 2 μm, and the arrows indicate the direction of the laser linear polarization. **b**–**d** Spatial distributions of one (**b**), two (**c**) and three (**d**) trapped Au NPs (400 nm in diameter) at the glass/solution interface. The trapping laser is linearly polarized in the y-direction. (Upper left): The two-dimensional distribution in the x-, y-focal plane and their trajectories, in x- and y-directions over time. As a visual aid, their colors correspond to each NP in the two-dimensional distribution. (Lower right): The localized distribution in x- and y- directions for the one Au NP system and the interparticle distance distribution between adjacent NP in two and three-NP systems. The dashed-grey lines are the Gaussian fitting curves to estimate the center value and its distribution width.

A few seconds after switching on the trapping laser, the first Au NP is trapped at the center of the focal spot with a standard deviation of around 25 nm (Fig. 2a, b/ Supplementary Movie 1). Each movie was recorded during 10 s with a frame rate of 100 fps to study the NP(s) motion in the few tens of millisecond time scale. The position of the focal spot was determined by imaging the back-reflection pattern of the trapping laser and fitting of the image with a 2D Gaussian function to determine the center of mass of the particle(s). For details, we refer to the materials section. When a second NP arrives at the focus, both NPs are linearly aligned perpendicular to the laser polarization (Fig. 2c/ Supplementary Movie 1). The mean value of the center-to-center distance between the two NPs (hereafter interparticle distance) is 775 ± 4 nm, which is similar to the laser wavelength in water (λ* = λ_laser_/n_water_ = 1064/1.33 ≈ 800 nm, hereafter the wavelength refers to the effective wavelength in medium). The Pearson correlation coefficient (PR) of their fluctuation motion was calculated to be 0.97 and 0.74 in the direction perpendicular and parallel to laser polarization, respectively. When the linear laser polarization is rotated to a certain angle, the linear alignment of the two NPs also rotates with the same angle (See Supplementary Information S3). For all angles, the spatial correlation is always the largest in the direction perpendicular to linear polarization. These characteristics show that the two Au NPs are optically bound as a single system, presenting a correlated motion in the direction of light scattering.

When a third Au NP is trapped, a three-NP linear alignment (later referred to as 3LA) is formed perpendicular to the linear laser polarization (Fig. 2d/ Supplementary Movie 1). The mean interparticle distance is around 800 ± 12 nm, slightly longer than for the two-NP case (2LA). In the three-NP case, the two NPs located at the edges of the 3LA receive a weaker gradient force because they are farther away from the center of the focus, explaining the longer interparticle distance. The NP’s motion is highly correlated in the x-direction (0.81–0.92), similar to the two-NP system, while a weaker correlation is observed for the y-direction (0.0–0.49). These findings are in line with previous studies about optical binding inside the irradiated region^30,31^.

### Four-nanoparticle system

**Fig. 3:**
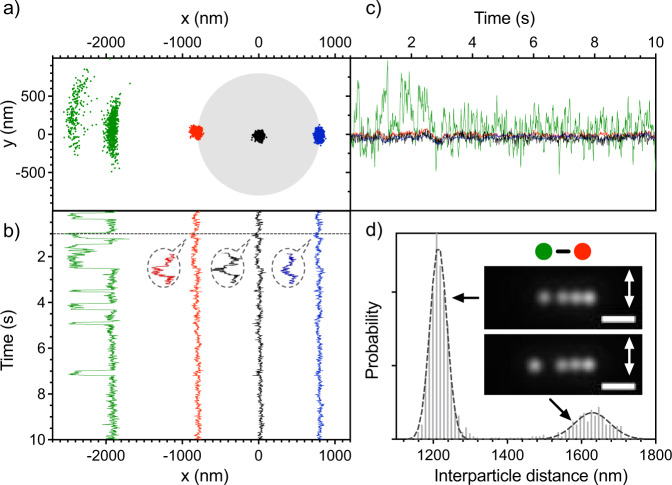
Spatial distributions for the four trapped Au NPs system. **a** The two-dimensional distribution in the x,y-focal plane, and the shadowed grey circle corresponds to the estimated diameter of laser focus (1.8 μm). **b**, **c** Trajectories of NPs in x- and y-directions over time, and their colors (black, red, blue and green) correspond to each NP in the two-dimensional distribution. The dashed-line bubbles show a magnification of the 3LA NP’s motion to show how they are affected by the external NP hopping. **d** Interparticle distance distribution between the external Au NP and the closest one inside the focus. The dashed lines are Gaussian fitting curves, which mean value approximately corresponds to 1.5 and 2 wavelength separations. Inset: Representative images when the external NP is localized at the first and second arc, respectively. The scale bar is 2 μm, and the arrows indicate the direction of the laser linear polarization.

The distance between the NPs located at the edges of the 3LA is roughly 1.6 μm. The focused laser beam diameter is estimated as 1.8 μm either by theoretical calculation or by experimental measurement (see further details in Supplementary Information S4)^32,33^, indicating that those NPs are located at the edge of the focus. Therefore, a fourth Au NP does not fit inside the focus, yielding a system with an external NP plus 3 NPs inside the laser focus (see Fig. 3/ Supplementary Movie 2). The external NP can be equally localized either on the right or left side of the focus. We only show the data when it is located on the left side to facilitate the discussion since the behaviour is mirrored-shaped when it is situated on the right side (see Supplementary Information S5).

Figure 3a–c shows that the external NP is localized in two discrete stable positions, whose spatial distribution resembles an arc. We term these positions first and second arcs based on their distance from the focal spot. The distance of the external NP’s to the closest one inside the focus is around 1.2 and 1.6 μm for the first and second arc, respectively (Fig. 3d). We note that these distances correspond to 1.5 and 2 times the laser wavelength in water. The NP motion in the x-direction (and to a minor degree for y-) outside the focus is also correlated with the NPs inside the focus. The correlation value is considerably larger when the external NP is localized in the first arc than in the second one (PR of 0.61 and 0.05, respectively). This finding indicates that the NP is more strongly bound with the 3LA in the first arc than in the second one (See Supplementary Information S6). Furthermore, we observe that the trajectory of the external NP (green line Fig. 3a–c) is more correlated with the closest particle inside the 3LA (PR of 0.66) than with the ones further away (PR of 0.60 and 0.52). Thus, the optical binding force between nearby NPs tends to be stronger when they are closer. Another aspect of this dynamics is that the whole 3LA slightly shifts to the left when the external NP hops to the second arc from the first arc and vice versa, showing small spikes in the trajectories (see the horizontal line and the magnified dashed-line bubbles in Fig. 3b). This implies that all the four Au NPs are bound as a single system, and therefore, the external NP movement affects the movement of the 3LA and vice versa.

The external NP hopping between arcs indicates that there exists a potential barrier between them (see the green line in Fig. 3a–c), which is low enough to be easily overcome by random thermal fluctuation. Besides, a larger potential barrier exists on the right side of the first arc, as can be deduced from its sharper borderline (see Fig. 3). Rarely, the external NP collides with the 3LA through a channel, which circumvents the potential barrier (see further details in Supplementary Information S7/ Supplementary Movie 3). In that case, the edge NP at the other side of the 3LA is pushed out from the focus like a Newton’s cradle system.

### Five-nanoparticle system

**Fig. 4:**
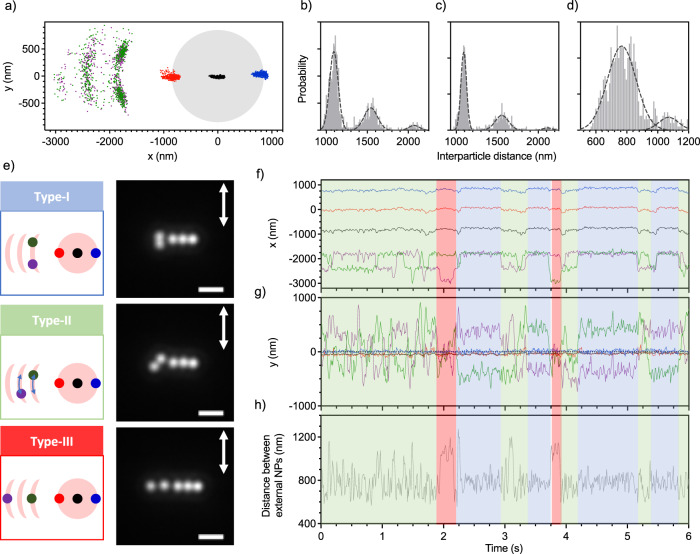
Spatial distributions for the five trapped Au NPs system. (**a**) The two-dimensional distribution in the x,y-focal plane, shadowed grey circle corresponds to the estimated diameter of laser focus (1.8 µm). **b**–**d** Interparticle distance distribution between (**b**) purple and red-marked NPs, (**c**) green and red marked NPs, and (**d**) green and purple marked NPs. The dashed lines are the Gaussian fitting curves which mean value approximately corresponds to 1.5, 2 and 2.5 λ* separations. **e** Illustrations and representative images for Type-I, Type-II and Type-III configurations. The scale bar is 2 µm, and the arrows indicate the direction of the laser linear polarization. **f**, **g** NP’s trajectories along the (**f**) x- and (**g**) y-directions over time. Note that each NP trajectory has the color code that corresponds to the NP’s position in (**a**). **h** Variation of the interparticle distance between the two external NPs overtime. The shaded blue, green and red regions correspond to the Type I, Type II, and Type III configurations, respectively.

Upon incorporation of a fifth NP, two of the particles are located outside the focus. Like the aforementioned four-NP system, the 3LA shows high-correlated motion (PR of 0.99 and 0.49 in horizontal and vertical directions, respectively). We have observed two different arrangements for the external NPs: i) one NP at each side of the focal spot or ii) two NPs at the same side. In this first arrangement, the external NPs present similar properties to the former four-NPs system. This is also the most common arrangement for the five-NP system. In the second arrangement, each external NP is bound with the 3LA, forming the arc-shaped distribution with a half-wavelength periodicity (Fig. 4a–d). Interestingly, the interparticle distance between the two external NPs is also discrete with a value of roughly 800 nm (1 λ*; Fig. 4d). Of note, a minor interparticle distance distribution is also observed around 1.0 to 1.2 μm (1.5 λ*; Fig. 4d). Overall, the two external NPs hop back and forth between the neighbouring arcs while maintaining specific alignments, which we classified into three different configurations (Fig. 4e) to further understand their dynamics (see Supplementary Movie 4).

In the Type-I configuration, the two external NPs are vertically aligned in the first arc or rarely in the second arc (Fig. 4f). Concretely, one NP is localized at the upper part of the arc, while the other one is localized at the lower part of the same arc (see illustration in Fig. 4e). The interparticle distance between both NPs is around 800 nm. This means that the two external NPs are not only optically bound with the 3LA, but also between themselves. We propose that the 3LA acts like a light source that constructively scatters the trapping laser from the focus to the external NPs. Then, the external NPs can scatter this light towards the other external NPs, facilitating a mutual interaction, which results in the formation of optical bounds outside the focus.

In the Type-II configuration, one NP is located at the upper part of the first arc, and another one is located at the lower part of the second arc, or vice versa. The external NPs are diagonally aligned with an interparticle distance distributed approximately 800 nm, which sometimes can lengthen up to 1.0–1.2 μm (close to 1.5 λ*; see illustration in Fig. 4e, h). The interparticle distance in the Type-II configuration fluctuates more than in the Type-I configuration, suggesting that the optical binding force between external NPs is weaker and/or disturbed by other forces. For instance, the electrostatic repulsive force between the external NPs is likely to play a role when the NPs are close. This is probably also why the external NPs are diagonally aligned. If they were aligned horizontally, the interparticle distance would be very short (≈400 nm), leading to large electrostatic repulsion.

Occasionally, we observed the Type-III configuration (Fig. 4e) in which one NP locates at the first arc while the other locates at the third arc, yielding an interparticle distance around 1.0 to 1.2 μm (1.5 λ*). The stability of Type-III configuration appears to be much lower than the other two configurations (compare red vs. blue or green shaded regions in Fig. 4f–h). Moreover, this is the only configuration we observed where the number of arcs reaches three (Fig. 4a).

These observations suggest that the external NP in the first arc can further scatter the light toward the outside assisting the binding of another external NP in the third arc. In other words, the scattered light is relayed by the external NPs, leading to a dynamic expansion of the optical potential outside the irradiated area. Although the external arcs are distributed along the direction perpendicular to linear laser polarization, the NP’s motion inside the arcs is parallel to laser polarization such as Type-I and II. Therefore, the laser polarization direction information is partially lost during multiple scattering processes among the external NPs.

### Six-nanoparticle system

When a sixth Au NP is trapped, the motion of each NP becomes much more complex and dynamic, and the number of possible configurations is dramatically increased as well. The stability of each configuration is smaller than in former reported systems due to the larger NPs mobility, leading to a more frequent rearrangement between the configurations. Thereby, the SPT analysis is more technically demanding, especially when the NPs exchange their positions, causing less accurate measurements of interparticle distance and correlation between NPs (see Supplementary Movie 5 for a representative case). Hence, in the six-NP system, we mainly describe the results based on the images of the different system configurations as well their spatial distribution.

**Fig. 5:**
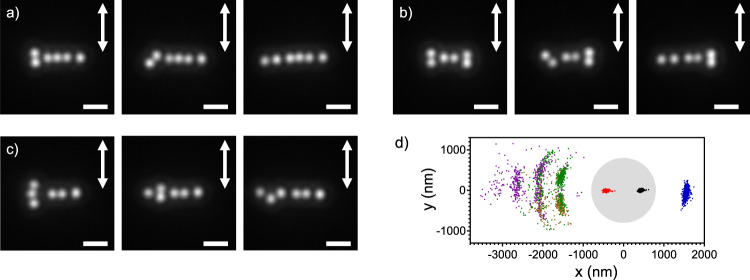
Scattering images and spatial distributions for different configurations of six trapped Au NPs. The number of NPs at the left side, center and right side are (**a**) 2:3:1, (**b**) 2:2:2 and (**c**) 3:2:1, respectively. The arrows indicate the direction of the laser linear polarization. The scale bar is 2 µm. **d** The x-/y- spatial distribution of the 3:2:1 arrangement of panel c experiment.

Figure 5 shows the observed configurations, which are classified by the number of NPs localized at the left side: center: right side respectively as (a) 2:3:1, (b) 2:2:2, and (c) 3:2:1. In this system, the external NPs are simultaneously distributed at both sides of the focus. The LA is composed by three or even two Au NPs. The 2LA is much more frequently observed in the six-NP system than in the former systems, where it is only scarcely observed. For example, one NP in 3LA hops outside the focus, and this sometimes happens when the external NP collides with the 3 LA. Depending on the number of external NPs at each side, we can treat this complex system as a combination of the above-mentioned four- and five-NP systems. Figure 5a shows that the 2:3:1 configuration can be simplified as a five-NP and four-NP systems for the left and right side, respectively. More specific, the two external NPs have similar dynamics as the five-NP system (e.g., the arc-shaped distribution and the abovementioned Type-I, Type-II, and Type-III configurations; see the three images in Fig. 5a). Likewise, the 2:2:2 configuration (Fig. 5b) can be simplified as two individual five-NP systems at each side. As in the former case, they can also present Type-I, Type-II, and Type-III configurations at both sides (see the three images in Fig. 5b). Noteworthy, in this case, the central alignment is composed of only two Au NPs; however, the scattered light from the 2LA is strong enough to stabilize such expanded configurations. Finally, the 3:2:1 configuration contains three external NPs at one side (Fig. 5c). Within this configuration, the external NPs can farther expand outside the focus. For example, the distance between the far-left NP and the nearest NP inside the focus is around 3 μm in Fig. 5c, which corresponds to the appearance of a fourth arc in the spatial distribution (Fig. 5d). It is worth mentioning that the fourth arc mainly appears in the 3:2:1 configuration, indicating that a larger number of external NPs lead to a further optical potential expansion. As such, it demonstrates the importance of the number of external NPs in the optical potential evolution.

## Discussion

In the results section, we thoroughly described the phenomena for one to six Au NP systems prepared with a 1064 nm laser at the upper glass/solution interface. Notwithstanding, one might consider that the observed optical binding can be (partially) induced (or enhanced) by the reflection of the trapping laser at a reflective interface. In order to study this possibility, we calculated the intensity of the reflected laser beam from the glass substrate according to both angular spectrum representation and Fresnel theory. The estimated reflected fraction is only 0.5% of the incident laser beam (Supplementary Information S8), suggesting a negligible role of light reflection under the optical conditions used in the experiment. To gain further confidence, we repeated the experiments, replacing the glass coverslip by quartz (*n* = 1.45) and sapphire (*n* = 1.75) coverslips, for which the reflection should be slightly smaller (0.2%) and larger (2.0%), respectively. We did not observe any major change on the optical binding properties (Supplementary Information S9), confirming that light reflection at the interface does not significantly contribute to optical binding under the condition used. Thus, we consider that the reflected light from the interface does not significantly contribute to optical binding either inside or outside the irradiated area. Therefore, as discussed before, the only role of the interface is to act as a physical barrier, at which the Au NPs can be gathered and stably trapped inside the irradiated area because both gradient and scattering forces contribute to generate a stable trapping spot. In contrast, inside the bulk solution, metallic NPs can only be trapped metastable using a large NA oil-immersion objective lens^9,34^. Hereafter, we will discuss the mechanism of optical binding inside and outside the laser focus which provides the arc-shaped Au NPs distribution. In addition, we explain how these few-particle systems evolve to the dumbbell-shaped swarming that we reported previously^26^.

The first incoming NP is simply trapped at the laser focus due to the gradient force which is caused by the interaction between the incident trapping laser and the induced polarization of NP. When two NPs are trapped by the gradient force, the NPs are polarized in phase by the incident laser because they are in the same laser focus. Meanwhile, the incident laser is partially scattered by one NP to the other NP and vice versa. Thus, both NPs mutually interact through the scattered light. The oscillation of induced polarization generated by the incident laser is affected by the scattered light depending on the separation between the NPs. Specifically, when the interparticle distance matches the wavelength, there is phase matching of the phase of the incident laser in one NP and the phase of the scattered light from the other NP, and therefore the induced polarization is constructively oscillating. As a result, these NPs experience an optical binding force (interparticle optical force) to find a stable position. The sign of optical binding force alternates from attractive to repulsive and vice versa in function of the wavelength periodicity. Thereby, the NPs eventually settle at a stable position where the repulsive force changes to attractive force, leading to the observed NP configuration at discrete wavelength intervals. Thus, the NPs inside the same laser focus are tightly bound as a single system due to the optical binding force. Moreover, the perpendicular alignment with respect to the polarization of the incident laser results from incident light being scattered perpendicularly due to dipole scattering. When the interparticle distance is not equal to a wavelength multiple, both NPs are depolarized because the incident and scattered light are not in phase. Such configuration is not energetically stable. This interpretation for two- and three-NP systems is in accordance with the conventional optical binding inside the laser focus.

Once the number of particles is above three, there is at least one external NP which is not directly illuminated by the laser and consequently, in first approximation, it cannot scatter light toward the 3LA. This first approximation contradicts our idea that the external particle(s) is (are) optically bound with the NP localized at the edge of the focus. Also, the external NP distributed in the arcs with half wavelength periodicity (1.5, 2, 2.5 and 3 λ*), cannot be explained by only considering direct illumination. However, our experimental results clearly show the arc-shaped distribution resembling a light scattering pattern as well as a correlation between the motion of the external NP and the 3LA. Therefore, we propose that the 3LA efficiently scatters the light to the external NP, and then the light is scattered back to the 3LA by the external NP. Accordingly, the 3LA and the external NP mutually interact through light scattering, triggering the optical binding even outside the focus.

The optical binding by a back-scattered light mechanism was early reported by Wei et al.^35^ Under their condition, two polystyrene microparticles were trapped using two independent lasers. Briefly, the light was scattered from particle A to particle B and is subsequently scattered back to particle A (A → B → A). In this case, the sign of the binding force (attractive to repulsive forces and vice versa) changes with a half-wavelength periodicity, indicating that local minimum potential positions appear at each half-wavelength distance. The optical path length from particle A to B to A is a multiple of the wavelength. Therefore, the phase of scattered backlight and incident trapping laser are the same, which satisfies the optical binding condition. Similarly, a longitudinal binding geometry in a counter-propagated laser beam also provides the half-wavelength periodicity due to the back-scattered light^11,36^. In these discrete periodic positions, the induced polarization of each NP has the same phase with the scattered light, which infers an extra stabilization energy providing the deepest optical potential. From this perspective, the half-wavelength periodic interparticle distance strongly supports that the external NP is optically bound with the 3LA by the back-scattered light. It should be mentioned that, based on these insights, the half wavelength interparticle distance, around 400 nm, represents a stable position for external NPs. However, considering the large particle size (400 nm in diameter), the NPs would be in direct contact, which is likely prevented by electrostatic repulsive forces. In addition, the arc at one wavelength distance is also not observed, however, this cannot be explained by the electrostatic interaction. We consider that the scattered light from the 3LA not only induces an optical binding force, but also, induces a scattering force, which pushes the external NP away from the focus. Indeed, this large scattering force also explains the right borderline of the first arc distribution (green distribution in Fig. 3a), as in detail stated in Supplementary Information S7.

In the five particle cases, the interparticle distance between two external NPs (green and purple) is around that of the trapping wavelength, indicating that the two external NPs are not only optically bound with the 3LA inside the focus, but also between themselves. In this case, one of the external NPs (green) receives light from the 3LA, and then the light is further scattered to the second external NP (purple). Meanwhile, there is also an optical pathway from 3LA to green to purple-colored external NPs. Such scattering light mutually couples the external NPs yielding an optical binding between them. The interparticle distance for type III configuration (one external NPs in the first arc and another in the third arc) is lengthened to around 1-1.2 μm, and the distance between each arc is found to be around 550 nm. We consider that these modifications are due to the optical force between the external NPs and the minor electrostatic repulsive force by the presence of a fifth NP. All in all, the two external NPs are distributed at energetically favourable configurations as the total system satisfies the optical binding condition among the three arcs. For the six-NP system, the event that the one NP hops from the 3LA without any collision suggests that the optical potential at the outside becomes relatively deeper due the presence of more external NPs compared to four and five-NP systems. Of note, the registered scattered intensity of the different NPs does not significantly fluctuate over the acquisition time, which indicates that their motion along the axial position is limited, yielding a 2D structure at the interface. This observation is consistent with the directionality of the scattering events of the central alignment, which constructively propagates along the interface and perpendicular with respect to the incident polarization^37^.

From these observations, we can deduce that the optical forces dominating these dynamics are i) the gradient force induced by the incident laser beam; ii) the optical binding force among the linearly aligned NPs in the 3LA; iii) the optical binding force between the 3LA and an external NP; and iv) the optical binding force between two (or more) external NPs. These forces lead to three different types of optical bounds: between two irradiated NPs, between one irradiated and one non-irradiated NP, and between two non-irradiated NPs. These two last forces are essential for forming optical matter outside the irradiated area through a multiple scattering mechanism.

We should consider that hydrodynamic coupling between particles can lead to collective motions which might potentially disturb the optical scattering pattern^38,39^. Something similar may also occur with convective or Marangoni flows arising from local temperature elevation. However, in the present condition, these effects are minor because they are not strong enough to effectively distort the optical binding properties. In previous work, we estimated a temperature increase of around 20 °C, when 3 Au NPs were printed onto the solution/glass surface and irradiated with a focused 1064 nm laser with the same power as used in this work^27^. Donner et al, calculated that the induced inward flow due to a similar heat elevation should be less than 10 nm/s^30,40^. In fact, the three NPs in the central alignment are strongly optically bound, and barely move, which implies that the hydrodynamic coupling and the thermal fluctuations enhancement are negligible and therefore their scattering pattern is stable. External NPs (either on the right or the left side of the central alignment) can move along this expanded optical potential, however, they also do not modify the main pattern/central alignment, as shown in Fig. 5d, ruling out a major effect of hydrodynamics couplings as well as the presence of convective or Marangoni flows. Nevertheless, we cannot rule out a minor contribution of the temperature on optical binding outside the irradiated area because the NPs show more dynamic motions inside the different arcs. This motion will become more vigorous when local temperature rises. Similarly, although the number of external NPs (3 at most) is small, and their distance is large enough to avoid strong hydrodynamic interactions^39^, weak coupling between them might create some minor correlation between the external NPs displacements along the expanded optical force field.

**Fig. 6:**
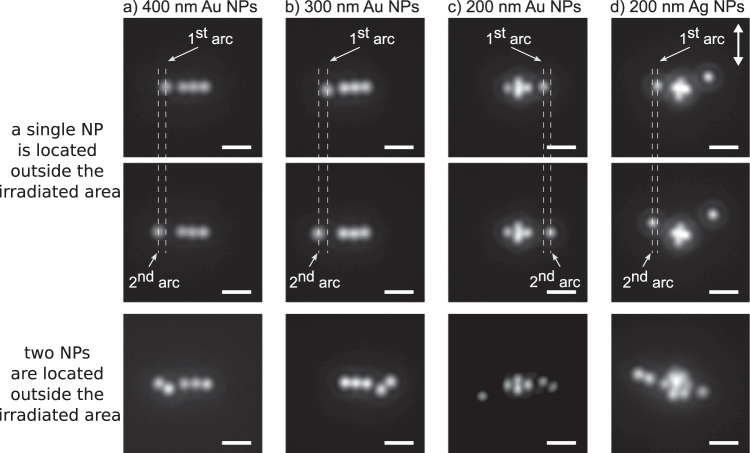
Representative scattering images, when 1 or 2 NPs are located outside the irradiated area. The white scale bar is 2 μm, and the white double-pointed arrow indicates the direction of the laser linear polarization. Used NPs: (**a**): 400 nm Au NPs, (**b**): 300 nm Au NPs, (**c**): 200 nm Au NPs, and (**d**) 200 nm Ag NPs.

Finally, one might wonder if the observed phenomena can be translated to other systems. In a first step, we repeated the experiments on Au NPs with a size of 300 and 200 nm (Fig. 6b, c, respectively). Similar as in the case of 400 nm Au NPs (Fig. 6a), the NPs are gathered outside the irradiated area in an arc-shaped distribution with an inter-arc distance of half the wavelength of the trapping laser, and only after the central alignment is arranged. Of note, when 200 nm Au NPs are used, more NPs are collected inside the focus along the direction parallel to laser polarization. The interparticle distance between two external (outside the focal area) NPs is also approximately that of the trapping laser wavelength. Thus, at a first glance, the only remarkable difference is that the motion of the external NPs is more dynamic for smaller NPs (Supplementary Movie 6). This observation is attributed to two factors: i) more vigorous Brownian motion due to the smaller NP size; and ii) the formation of weaker optical binding potential outside the irradiated area as the scattering cross section of the Au NPs decreases with the NP size^28^. As a consequence, the overall scattering intensity from the central alignment is reduced, leading to weaker optical binding outside the irradiated area. In the limit, Brownian fluctuation will overcome the optical binding force, blocking the expansion of the optical potential outside the irradiated area as happens when 100 nm Au NPs are used^28^. In a second step, we repeated the experiments using 200 nm silver NPs as another example of a metallic material with a SPR band (Fig. 6d). The Ag NPs also show the formation of optically bound structure outside the irradiated area and the NPs dynamics seem to be similar to the ones of 200 nm Au NPs. Instead, these observations are not seen when we replace metallic NPs with polymeric NPs (as a representative example of dielectric particles), indicating that the generation of optical binding outside the irradiated area with the same working principle is not possible. However, optical binding outside the focus for dielectric objects is still possible based on a light propagation mechanism as we previously demonstrated^41^. Taken together, these experiments reveal that the formation of optically bound matter outside the irradiated area requires a large flux of scattered or propagated photons.

With all this information in mind, we can now rationalize how these primary systems evolve to the swarming. By increasing the number of NPs, more optical pathways will become possible, leading to more varied configurations while keeping discrete half-wavelength interparticle distances. The rearrangements of the NPs in these systems will also become more frequent and arbitrary because the possible optical binding conditions arise non-linearly with the number of particles. This will lead to an enlarged optical potential by expanding the trapping laser effective range through multiple scattering processes. The generated optical potential outside the focus is shallower for the distant arcs, yielding a more erratic NP motion as well as facilitating the NPs hopping between arcs, both driven by random thermal fluctuations. If the number of particles keeps increasing, the optical potential will gradually smoothen to become a continuum, which explains well the previously observed dynamically fluctuating dumbbell-shaped swarming assembly, covering an area up to about 150 um^2^ (20 and 8 um for length and width, respectively)^26^. Under this condition, the hydrodynamic interactions will become relevant as they scale super-linearly with the NP density^38,39^. We suggest that most of the NPs in the system are optically interacting and bound by multiple light scattering events synchronized with hydrodynamic collective effects. In this way one can control the extend of binding outside the irradiated area. As multiple consecutive scattering events exist, the scattering light directionality along the interface is partially lost, potentially resulting in assemblies with a 3D morphology. Hence, the dynamically fluctuating swarms of Au NPs results from the light scattering interaction of the NPs throughout the system.

The presented results pave the way to prepare periodic arrays of metallic NP to act as optical nanoantenna and/or plasmonic crystal solely by focusing a trapping laser at the interface of a metallic NP suspension. Therefore, even though our data show large motion fluctuations, particularly when a large number of NPs are involved, the preparation of such structures should be possible, considering that the stability of those arrays will be determined by two factors. The first is the strength of the optical binding which can be enhanced by tuning the trapping laser wavelength to precisely excite the SPR dipole mode of the NP, increasing the scattering cross section, while not significantly enhancing the heat release^28^. Stronger optical binding can also be generated, if the scattering efficiency of the overall central alignment is increased. For instance, by using a widefield laser to increase the number of gathered NPs at the central alignment and therefore increase the overall scattering ability. Alternatively, the optical binding strength can be enhanced by a partial reflection of the trapping laser at the interface^18^. The second factor is to minimize the thermal Brownian fluctuations by increasing the viscosity and/or decreasing the temperature of the surrounding to efficiently dissipate the heat generated by the photoexcited NPs^27^. On the other hand, the formation of a more flexible geometry could be potentially achieved by controlling the direction of the optical binding force, which is essentially the same direction as the light scattering and can be controlled by rotating the laser polarization (Supplementary Information S10). Otherwise, the use of designed nano-antenna nanopatterns, that can act as nano-antenna, or favoring a specific arrangement of the central alignment NPs could yield a more flexible geometry of the optically bound matter outside the focus^42^. The formation of such periodical alignments can further change the optical properties of the overall system due to the multiple scattering/far-field interaction^43–49^. As an example, it was reported that the reflection maximum of an array of silver nanorods (200 nm in diameter) can be tuned from about 750 nm to 1300 nm solely by changing the array periodicities from 400 nm to 700 nm (center to center distance)^50^. The current work demonstrates that similar NPs arrays can be prepared through optical trapping; and therefore, it should be possible to optically-induce organized devices such as a reflection mirror or a diffraction lens by tuning the trapping laser wavelength. Moreover, 3D optically bound structures may be generated by using a counter-propagating laser beams, which can generate a 3D central alignment and an eventual resulting optical binding network with a 3D configuration. We believe that our finding is a critical step to understand the light-matter interaction in the evolving assemblies and will pave a new way for optical manipulation outside the focal spot.

## Methods

### Optical setup

The optical trapping system is constructed on an inverted microscope (Fig. 1). A 1064 nm continuous wave laser is tightly focused approximately 1-2 μm inside the upper glass/solution interface by an air-immersion objective lens (NA 0.90, 60x, Olympus UPlanFLN 60X). The laser power after the objective lens is set to 20 mW. The diameter of the laser is about 1.5 µm at the focal plane (see Supplementary Information S4). A half-wave plate is used for rotating the direction of linear laser polarization. A halogen lamp illuminates the sample through an oil-immersion dark-field condenser lens (NA 1.2-1.4; Olympus). The scattered light by the trapped Au NPs is collected by the objective; filtered by a short-pass optical filter (Semrock, FF01-1010/SP-25) to remove the 1064 nm laser backscattered light; and recorded using a scientific Complementary Metal–Oxide–Semiconductor (sCMOS) camera (100 fps).

### Sample preparation

The sample was prepared by sandwiching 10 µl of a 400 nm Au NPs colloidal suspension (1.9 × 10^5^ particles/mL, Sigma-Aldrich) between two clean coverslips with a 120 µm depth spacer (Electron Microscopy Sciences). Before sample preparation, the Au NPs suspension was sonicated for 10 min to disperse the NPs. The coverslips were cleaned using an ozone treatment (60 min) to avoid the adhesion of the Au NPs to the glass substrate.

## Supplementary information

Supplementary Information

Description of Additional Supplementary Files

Supplementary Movie 1

Supplementary Movie 2

Supplementary Movie 3

Supplementary Movie 4

Supplementary Movie 5

Supplementary Movie 6

## Data Availability

The localized positions of gold nanoparticles obtained from the particle tracking analysis (in Figs. 2–5) are provided in the Source Data file. The relevant movies are provided as Supplementary Movies. Additional supporting data are available upon reasonable requests to the corresponding author. Source data are provided with this paper.

## Source data

Source Data

## Code availability

The developed code for particle tracking and its subsequent analysis is available at the following link: https://github.com/BorisLouis/goldTracking/releases/tag/v1.0.
