# Supplementary material for: The primeval optical evolving matter by optical binding inside and outside the photon beam
Source: Nat Commun. 2022 Sep 10;13:5325. doi: 10.1038/s41467-022-33070-w (PMC9464242; doi:10.1038/s41467-022-33070-w)
Supplement: Supplementary file 1 — Supplementary Information [file 41467_2022_33070_MOESM1_ESM.pdf]

The primeval optical evolving matter by optical binding inside and outside the photon beam

Author list:

Chih-Hao Huang[1,+], Boris Louis[2,3,+], Roger Bresolí-Obach[1,2,4*], Tetsuhiro Kudo[1,5*], Rafael Camacho[2,6], Ivan G. Scheblykin[3], Teruki Sugiyama[1,7,8*], Johan Hofkens[2,9*] and Hiroshi Masuhara[1,8*]

[+]These two co-authors contributed equally

Corresponding authors: roger.bresoliobach@kuleuven.be, kudo@toyota-ti.ac.jp, sugiyama@g2.nctu.edu.tw, johan.hofkens@kuleuven.be, masuhara@masuhara.jp

Affiliations:

1. Department of Applied Chemistry, College of Science, National Yang Ming Chiao Tung University, Hsinchu 30010, Taiwan.

2. Laboratory for Photochemistry and Spectroscopy, Division for Molecular Imaging and Photonics, Department of Chemistry, Katholieke Universiteit Leuven, Leuven 3001, Belgium.

3. Division of Chemical Physics and NanoLund, Lund University, Lund 22100, Sweden.

4. Department of Analytical and Applied Chemistry, Institut Químic de Sarrià, Barcelona 08017, Spain.

5. Laser Science Laboratory, Toyota Technological Institute, Nagoya 468-8511, Japan.

6. Center for Cellular Imaging, Core Facilities, the Sahlgrenska Academy, University of Gothenburg, Gothenburg 405 30, Sweden

7. Graduate School of Materials Science, Nara Institute of Science and Technology, Nara 630-0192, Japan.

8. Center for Emergent Functional Matter Science, National Yang Ming Chiao Tung University, Hsinchu 30010, Taiwan.

9. Max Planck Institute for Polymer Research, Mainz 55128, Germany.

S1. Tracking code

The code used in this work for tracking is in majority based on the code described in our previous publication.[1] However, in this case, we took advantage of the fact that we knew exactly how many particles are trapped in each system. Therefore, we made a forced detection of n number of particles by detecting the brightest pixel on the image followed by a deletion of the said pixel and the one surrounding it. We repeated these steps until n particles were detected. After that, n gaussian were fitted simultaneously to the image to optimize the accurate localization of the particle positions. Finally, we compared subsequent frame while minimizing the sum squared of displacement to perform the tracking. The algorithm is available at https://github.com/BorisLouis/goldTracking

S2. 400 nm Au NPs swarming formation

In the Main text, we imaged the motion of optically trapped Au NPs (one to six) using a sCMOS camera (100 fps) for studying their motion in detail. Here, a supplementary experiment was carried out in which we increased 200-fold the Au NPs concentration to observe the swarming assembly formation. For this experiment, the sCMOS camera was replaced by a CCD camera (30 fps) for long time recording. Upon switching on the 1064 nm trapping laser, the Au NPs are gradually trapped at the upper glass/solution interface, forming a linear alignment with three NPs inside the focus (Fig. S1a-b). After a while, more Au NPs are trapped outside the focal spot. As described in the Main text, they are optically bound between each other, presenting a dynamic fluctuation motion. Stochastically, they can rearrange into different configurations. For example, in the five-NP system, the external Au NPs can distribute either one NP at each side (Fig. S1c) or both NPs at the same side (Fig. S1d). When more NPs come, their motion becomes more dynamic and erratic, which leads to a more frequent rearrangement between configurations. Eventually, two swarms of Au NPs are formed in the direction perpendicular to linear laser polarization, leading to the previously described dumbbell-like swarming assembly (Fig. S1f).[2] Of note, the system's complexity (e.g. the appearance of several different configurations, which probability is low) rises upon increasing the particle number. For example, Fig. S1f shows a configuration with 4 Au NPs in the central alignment (1:2:1), which is never observed in the initial stage.

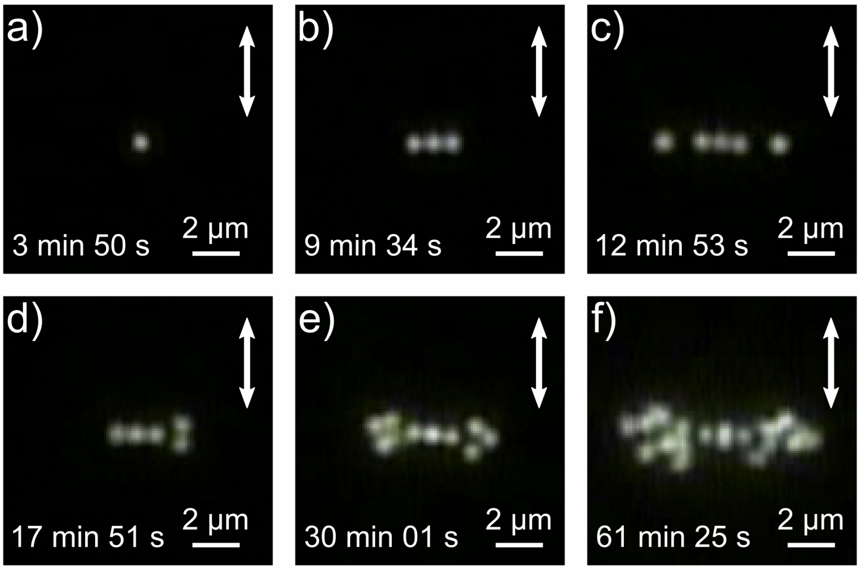

Supplementary Figure 1. Scattering images of dumbbell-like Au NPs swarming assembly formation prepared by 1064 nm laser trapping for different laser trapping irradiation times: (a) 3:50 min; (b) 9:34 min; (c) 12:53 min; (d) 17:51 min; (e) 30:01 min; (f): 61:25 min. The Au NPs diameter is 400 nm; its concentration is 3.8 x 10^7 NPs/mL, and the laser power is 20 mW. The laser irradiation time is indicated in each image. The white double pointed arrows refer to the direction of linear polarization, and the scale bar is 2 μm.

S3. The highly correlated motion of the optically bound Au NPs

To further study the impact of laser polarization on the observed correlated motion, we performed the following experiment in the two-NP system. In this configuration, both NPs are located inside the focal spot, and they are optically bound with each other. The trajectories of each NP are tracked by single particle tracking analysis. Later, we calculate the Pearson correlation coefficient (PR in the Main text) from the obtained traces. As described in the Main text, the NPs motion is highly correlated in the direction perpendicular to the linear laser polarization. Simultaneously, this correlation is reduced in the orthogonal direction (parallel to linear laser polarization).

To confirm this, we systematically rotated the laser polarization and examined the correlation coefficient in x- and y-direction, as shown in Fig. S2. The correlation coefficient is nearly one in y-direction when the laser polarization is centered at the x-direction, and vice-versa. Concretely, the PR shows a sinusoidal oscillation dephased by 90° with respect to the linear laser polarization in x- and y- directions.

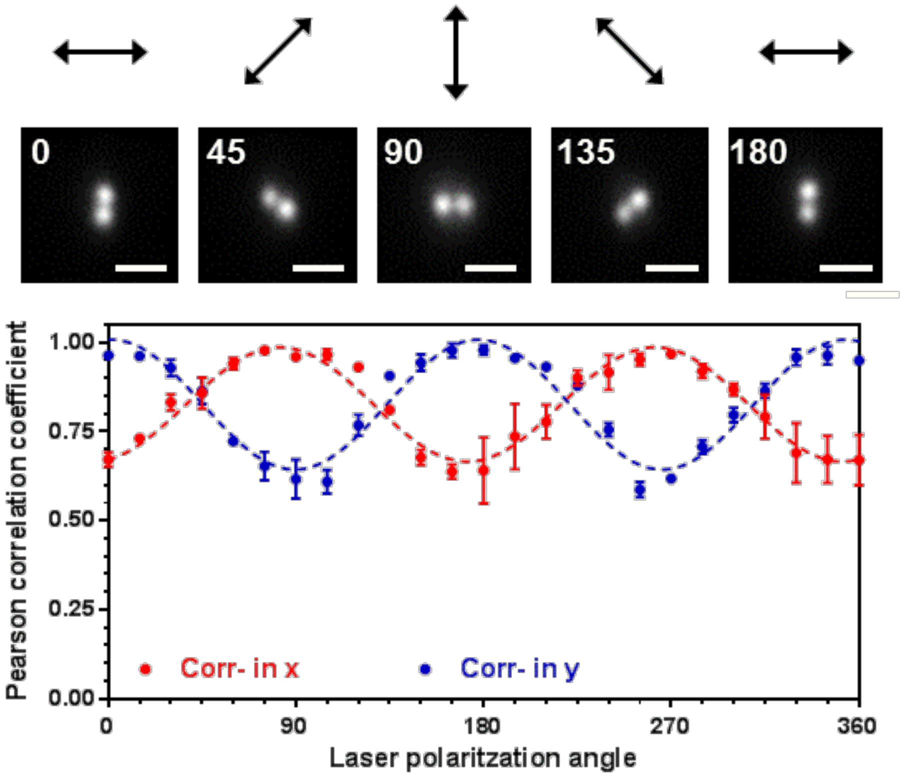

Supplementary Figure 2. Changes in the Pearson correlation coefficient (PR) for the motion of two trapped Au NPs upon rotating the linear laser polarization. Top: Scattering images for the two-NP system using different linear laser polarization directions. The black arrows refer to the direction of linear laser polarization, and the scale bar is 2 μm. Bottom: Pearson correlation coefficient of the NPs motion for the x- and y-direction with different linear laser polarization directions. The Pearson correlation coefficient is calculated and plotted in the x- and y-direction separately for a total of 360 degrees. The error bars refer to their standard deviation.

S4. Estimating focal spot size from NA value

Here, we theoretically and experimentally estimate the focal spot size near the interface. As the laser is focused approximately 1-2 μm above the upper glass/solution interface in the present experiment, we first calculated the intensity of tightly focused laser beam near the glass/solution interface by using the method of angular spectrum representation.[4] In the calculation, the light consists of various incident angle (k-vector spectrum or angular spectrum) is interfered at the focus, and the reflection from the glass/solution interface is included. Figure S3 shows the intensity profile at the interface when the laser is focused 1.5 μm above the interface (See Scheme 1 in Main text). The focal diameter (defined by $1/e^2$ width) along the x-axis is approximately 1.8 μm (Fig. S3b). The x-axis is the direction perpendicular to the linear polarization and the three-NP linear alignment (3LA) is formed in this direction as we explained in Main Text. This length is enough to accommodate up to three NPs and fourth NP is populated outside the focus.

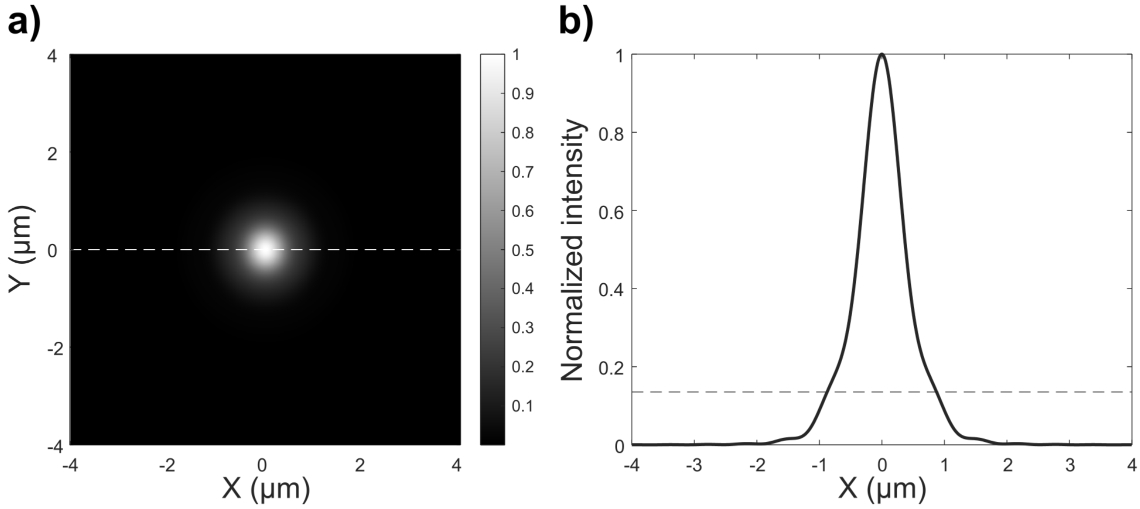

Supplementary Figure 3. a) Calculated intensity distribution of the tightly focused laser beam. The optical parameters are identical to the experimental conditions, the light is polarized in y-direction. The laser is focused 1.5 μm inside the glass/solution interface and the laser field at the interface is showed here in the left side. The profile along x-direction across the center (dashed line) is plotted as the right graph. b) Normalized intensity cross-section along x-direction. The horizontal dashed line indicates the value of $1/e^2$.

Next, we experimentally obtained the back-scattering image of the used trapping laser with our home-build multiplane widefield microscope (Fig S4).[1] The multiplane widefield microscope can simultaneously image up to eight different planes. Therefore, we aligned the trapping laser at the first imaging plane, and we imaged the back-reflection of the trapping laser at the fourth imaging plane, which corresponds approximately to a depth of 1.5 μm below the first imaging plane. A diameter ($1/e^2$ width) of 1.7 and 2.2 μm is obtained for the x- and y- directions respectively.

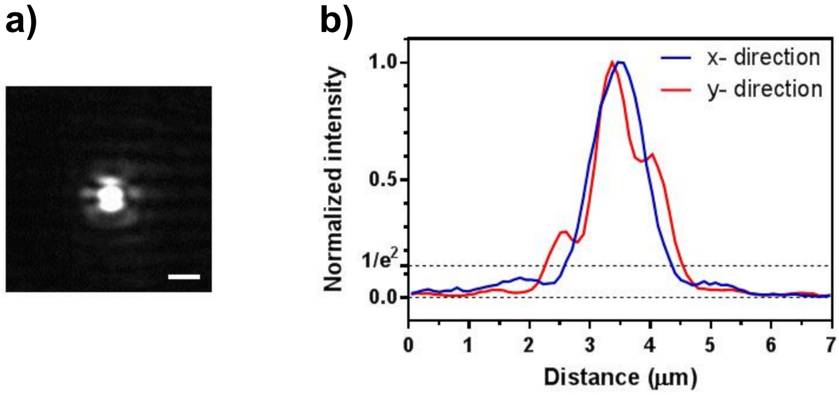

Supplementary Figure 4. Experimental determination of the laser beam size. a) back-reflection image from the trapping laser at the optical conditions used in this work. b) Normalized laser intensity profile along the x- and y- directions. The scale bar is 2 μm, and the horizontal dashed line indicates the normalized intensity value of $1/e^2$.

However, the trapping laser profile using the back-reflection images is convoluted with the microscope system's point spread function and can be modified to some extent by its partial refraction at the interface and the dichroic mirror. Thereby, we defined the focal diameter in Main text (such as shadowed grey circle in Fig. 2, 3 and 4) as 1.8 μm which is theoretically derived.

S5. The overall symmetry of the external Au NP

As explained in the Main text, the three-NP linear alignment (3LA) fully occupy the focal spot. Therefore, when the fourth NP enters the system, it is located outside either at the left (Fig. S5a) or the right side (Fig. S5b). The external NP moves much more dynamically than the NPs inside the 3LA, and it can shift between two sides via rearrangements. Occasionally, the four-NP system shows a rare case in which two Au NPs are trapped inside the focal spot, and the other two NPs are located outside, one at each side (Fig. S5c).
No matter how many Au NPs are in the system, the resulting assemblies tend to distribute symmetrically. In this work, we choose the cases where the external Au NPs are located only at the left side of the focus in order to focus on the discussion of optical binding between external NPs. Such a condition is easy to find for systems containing up to five NPs. After the sixth NP comes, it is difficult to have the three external NPs located on the same side for a long time (> 5s). In total, the Au NPs assemblies formed by laser trapping show a global symmetry over time, and this is plausible because the trapping laser is, of course, irradiated at the interface homogeneously.

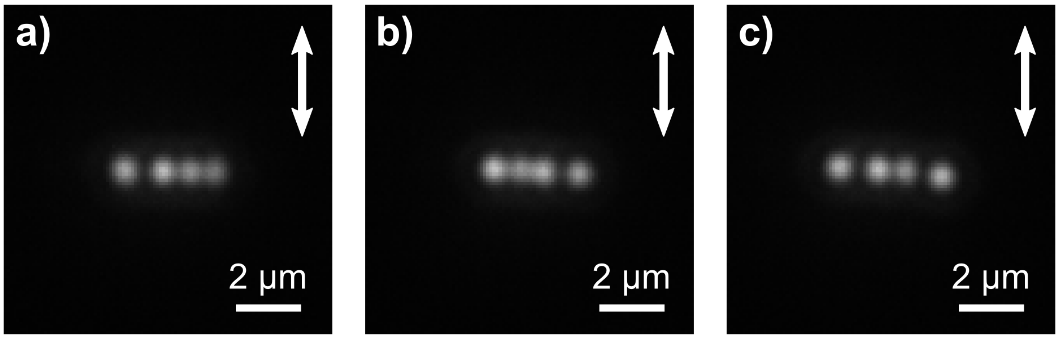

Supplementary Figure 5. A representative image for each configuration observed in the four-NP system. (a): three-NP linear alignment in the focus and one Au NP in the left side outside the focal spot. (b): three-NP linear alignment in the focus and one Au NP in the right side outside the focal spot. (c): two-NP linear alignment in the focus and two Au NPs outside the focal spot; one at each side.

S6. Comparison of the correlation coefficient at the first and second arc

To evaluate how the motion of the external NP is correlated with the motion of the 3LA, we calculated the Pearson correlation coefficient (PR) between the motion of the external NP (green dots) and the nearest NP inside the 3LA (red dots) among the x-direction. During the experiment's time course (10 s), the external NP hops several times between the first and second arc, as shown in green line of Fig. S5a-(ii). To discuss the correlation when the external NP is located at different arcs, we defined the boundary between the first and the second arc at x = -2200 nm (the horizontal dashed line in Fig. S6a-ii). Interestingly, the PR is 0.61 and 0.05 when the external NP is located at the first (Fig. S6b) and the second arc (Fig. S6c), respectively.
We rationalize this finding as both NP movements should be more synchronized since they are bound as a single system for larger optical binding forces. Therefore, we consider that the PR value is related to the optical binding force's strength. The result suggests that the external NP is optically bound with the 3LA, and this binding force is significantly stronger when the external NP is located at the nearer arc.

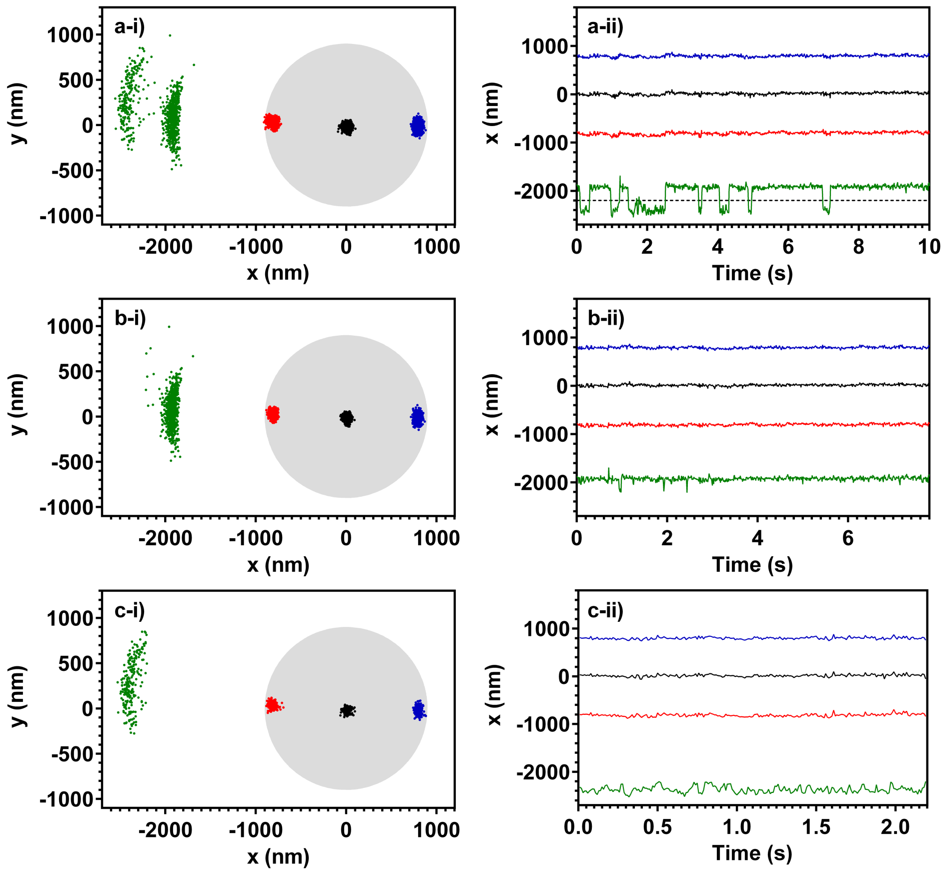

Supplementary Figure 6. The spatial distribution and the fluctuation in the x-direction for the four-NP system. (a) All the measured data in one typical experiment (10 s; 1000 frames). The data is separated into (b) and (c) according to the position of the external NP at the first and second arc, respectively. Practically, the boundary between the first and second arc is at -2200 nm (horizontal dashed line in a-ii).

S7. The potential barrier between the first arc and the focal spot

As observed in Figure 2 of the Main text (the four-NP system), the external NP can dynamically hop between the first and second arc. The external NPs frequently rearrange their positions and change their relative positions. However, the NP hopping seems to be hampered from the first arc to the 3LA. Specifically, a clear boundary is observed at the right side of the first arc, indicating a large potential barrier that hampers the external NP enter to the 3LA.
Figure S7 shows the sequential images of one rearranging process for a four-NP system (see Supplementary Movie S3). Before the rearrangement, the external NP dynamically fluctuates inside the two arcs and hops between them. Then, the NP moves toward one edges of the first arc (in this case, downside; Fig. S7a-c) and immediately enters the 3LA (i.e. inside the focus; Fig. S7d). The four Au NPs squeeze inside the focal spot, forming an unstable configuration for a short period (Fig. S7e). The 3LA is recovered by kicking out the NP at the right side toward the outside (Fig. S7f). Of note, the external NP tends to enter the focus through a channel with a vertical shift, trying to circumvent the high potential barrier induced by the scattering force from 3LA. The external NP has more chances to overcome the potential barrier when it is shifted away from the scattered light direction. The displacement from Fig. S7c to S7d is longer than before, implying that the external NP quickly falls into a potentially stable position once it overcomes the potential barrier.

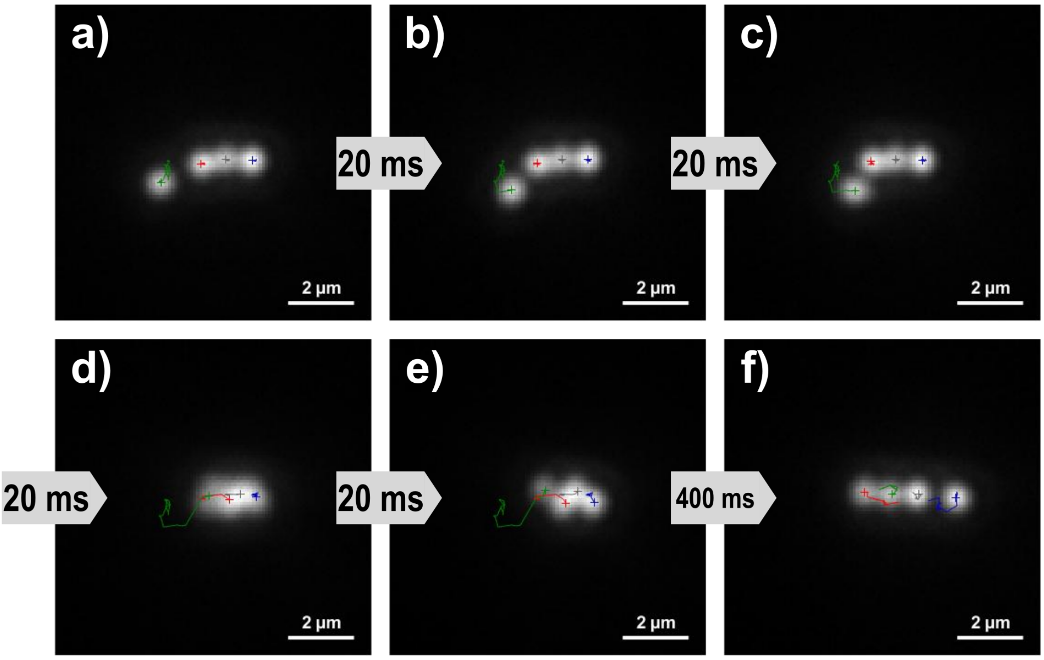

Supplementary Figure 7. Sequential scattering images of a rearranging process in four-NP system. The time interval between the images is indicated in the arrows. The times for the different images are the following ones: (a) 0 ms; (b) 20 ms; (c) 40 ms; (d) 60 ms; (e) 80 ms; (f) 480 ms. The traces of each Au NPs are marked with different colors. The scale bar is 2 μm.

S8. Estimation of the reflectance from the glass substrate

Depending on the incident angle and polarization of the trapping laser, the transmission and reflection of a photon beam can be described by the Fresnel coefficients at an interface. Figure S8 shows the calculated transmittance (blue) and reflectance (red) of the water-glass interface when light goes from water (n= 1.33) to glass (n = 1.518). The reflectance values for incident angles smaller than 43 degrees (the maximum possible incident angle due to NA of the objective) are mainly below 1%. Indeed, as the incident laser is tightly-focused, the average incident angle will be smaller than 43 degrees. Using the angular spectrum representation, which considers a tightly-focused laser profile, the incident polarization and the Fresnel coefficients, we estimated that the reflection on the water-glass interface is only 0.46%.

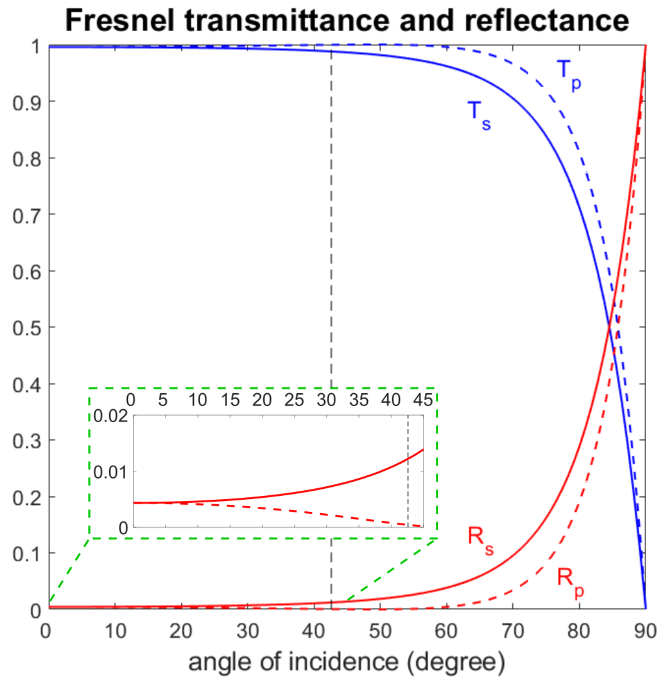

Supplementary Figure 8. Fresnel transmittance and reflectance when the light propagates from water (n = 1.33) to glass substrate (n = 1.518). The solid and dashed blue curves refer to the Fresnel transmittance of s-polarized (T_s) and p-polarized (T_p) incident light; while the solid and dashed red curves refer to the Fresnel reflectance of s-polarized (R_s) and p-polarized (R_p) incident light. The vertical black dashed line corresponds to the maximum possible incident angle (43°) considering the NA (0.90) of the objective. The green dashed inset is the enlarged plot of the Fresnel reflectance before 45 degree incident angle.

S9. Impact of the material interface on the optical binding properties

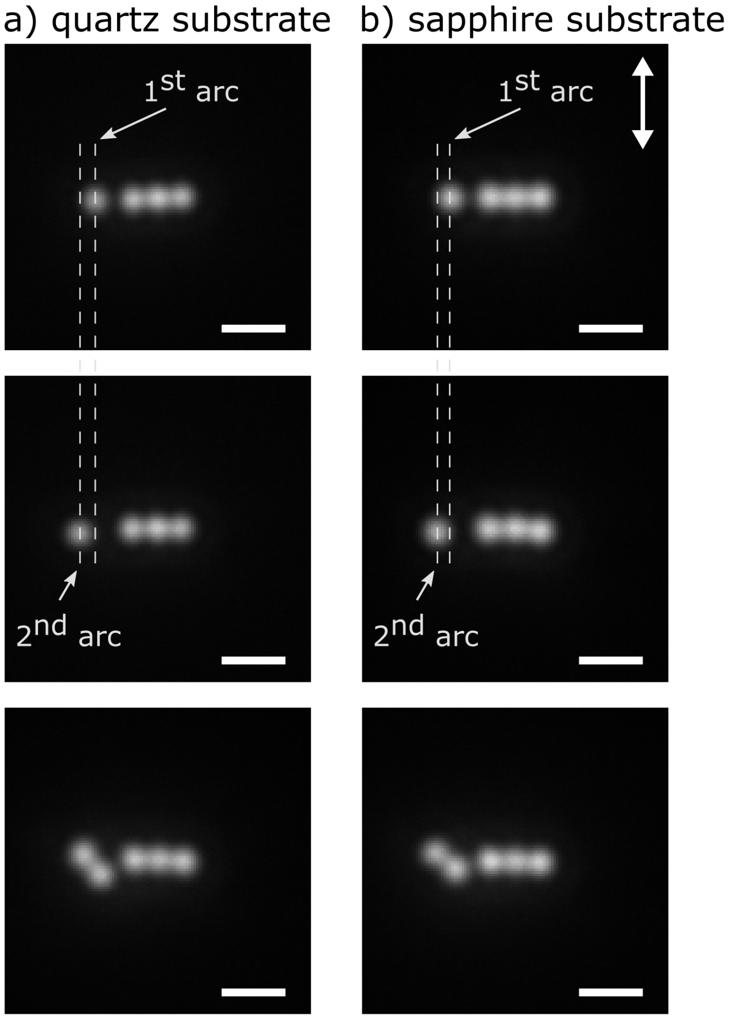

Supplementary Figure 9. Representative scattering images, when 1 (first and second rows) or 2 (third row) NPs are located outside the irradiated area. The white scale bar is 2 μm, and the white double pointed arrow indicates the direction of linear polarization. The assemblies are formed at a) quartz/solution interface and b) sapphire/solution interface.

S10. Impact of the laser polarization direction on optical binding properties

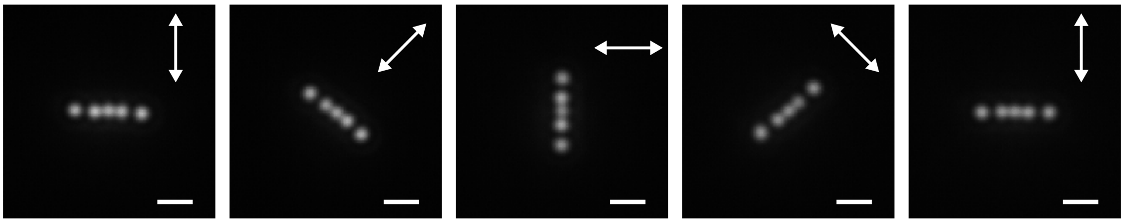

Supplementary Figure 10. The scattering images of 400 nm optically bound Au NPs for different directions of the linear polarization of the trapping laser. The overall NPs configuration is 1:3:1 (external left side: central alignment: external right side) which shows the directionality of the system with respect to the linear polarization of trapping laser. The white scale bar is 2 μm, and the white double pointed arrows indicate the direction of linear polarization.

S11. Supplementary References

(1) Louis, B.; Camacho, R.; Bresolí-Obach, R.; Abakumov, S.; Vandaele, J.; Kudo, T.; Masuhara, H.; Scheblykin, I. G.; Hofkens, J.; Rocha, S. Fast-Tracking of Single Emitters in Large Volumes with Nanometer Precision. *Opt. Express* **2020**, *28*, 28656-28671.

(2) Kudo, T.; Yang, S. J.; Masuhara, H. A Single Large Assembly with Dynamically Fluctuating Swarms of Gold Nanoparticles Formed by Trapping Laser. *Nano Lett.* **2018**, *18*, 5846–5853.

(3) Abbe, E. Beiträge Zur Theorie Des Mikroskops Und Der Mikroskopischen Wahrnehmung. *Arch. für Mikroskopische Anat.* **1873**, *9*, 413–468.

(4) Novotny, L.; Hecht, B.*Principles of Nano-Optics Second Edition*; Cambridge University Press: Cambridge, **2006**, pp.70-75
