# Supplementary material for: The primeval optical evolving matter by optical binding inside and outside the photon beam
Source: Nat Commun. 2022 Sep 10;13:5325. doi: 10.1038/s41467-022-33070-w (PMC9464242; doi:10.1038/s41467-022-33070-w)
Supplement: Supplementary file 2 — Description of Additional Supplementary Files [file 41467_2022_33070_MOESM2_ESM.docx]

**Description of Additional Supplementary Files**

**Supplementary Movie 1:** Representative dark-field movies for the one- (left), two- (center) and three- (right) Au NPs systems trapped at the upper solution/glass interface. The visualization speed has been slowed down by 10 times. The scale bar is 2 µm and the white arrow indicates the direction of trapping laser polarization.

**Supplementary Movie 2:** Representative dark-field movies for the four-Au NPs systems trapped at the upper solution/glass interface. The visualization speed has been slowed down by 10 times. The scale bar is 2 µm and the white arrow indicates the direction of trapping laser polarization.

**Supplementary Movie 3:** Representative dark-field movies for the colliding event observed in four-Au NPs systems. The visualization speed has been slowed down by 20 times. The scale bar is 2 µm and the white arrow indicates the direction of trapping laser polarization.

**Supplementary Movie 4:** Representative dark-field movies for the five-Au NPs systems trapped at the upper solution/glass interface. The visualization speed has been slowed down by 10 times. The scale bar is 2 µm and the white arrow indicates the direction of trapping laser polarization.

**Supplementary Movie 5:** Representative dark-field movies for the six-Au NPs systems trapped at the upper solution/glass interface. The visualization speed has been slowed down by 10 times. The scale bar is 2 µm and the white arrow indicates the direction of trapping laser polarization.

**Supplementary Movie 6:** Representative dark-field movies when two external NPs are optically bound outside the focal spot for a) 400 nm, b) 300 nm and c) 200 nm Au NPs.
